# Physical Properties of Ovalbumin/Sodium Carboxymethyl Cellulose Composite Gels Induced by Glucono-δ-Lactone and Heat Treatment

**DOI:** 10.3390/gels11100779

**Published:** 2025-09-29

**Authors:** Xiaofan Zhang, Lala Li, Liye Wang, Wei Xu

**Affiliations:** 1 College of Food and Drug, Luoyang Normal University, Luoyang 471934, China; zhangxiaofan@lynu.edu.cn (X.Z.); liye2009314@163.com (L.W.); 2 College of Life Science, Xinyang Normal University, Xinyang 464000, China; 15517984382@163.com; 3 Dabie Mountain Laboratory, Xinyang 464000, China

**Keywords:** ovalbumin, composite gels, Sodium carboxymethyl cellulose, rheological properties

## Abstract

In this paper, the effect of sodium carboxymethyl cellulose (CMC-Na) on the structure and gel properties of ovalbumin (OVA) gels induced by glucono-δ-lactone (GDL) and heat treatment was investigated. The results suggested that the interaction between CMC-Na and OVA was mainly through hydrogen bonding. The water-holding capacity of CMC-Na/OVA composite gels gradually increased as CMC-Na concentration increased, while the viscoelasticity was first enhanced and then weakened. The hardness and elasticity of the composite gels were higher than those of the pure OVA gels when the CMC-Na concentration was lower than 0.4%. However, the hardness and springiness of the composite gels decreased significantly after further increasing the CMC-Na addition. The change in texture properties induced by CMC-Na under glucono-δ-lactone (GDL) and heat treatment provided theoretical support for developing soft gel products with abundant protein for special groups, such as the elderly, teenagers, and pregnant women.

## 1. Introduction

Ovalbumin (OVA) is an easily available, inexpensive, and nutritious animal protein [1]. It has functional properties, such as gelation, foaming, emulsification, and water-holding properties, and has become an important food ingredient [2,3]. Among them, OVA gel is obtained by protein denaturation and aggregation to form a continuous network structure. Its textural characteristics are modulated by many factors, such as pH, heat, and salt ion concentration. Researchers have found that OVA gel can be formed by using heat-induced OVA aggregation or adding glucono-δ-lactone (GDL) as an acidifying agent. Different alkalinity could also impart desirable properties to ovalbumin (OVA), such as improving gel texture [4,5,6]. However, the stability of OVA gel is poor, which limits its application in special group foods. Many methods have been used to regulate the gel properties. Some flavonoids, such as naringenin, genistein, and naringin, were found to promote the gelation of low concentration and strength of ovalbumin gel [7].

Recent studies have shown that polysaccharide addition is a potentially effective strategy to modulate protein gel performance. For example, the gelling properties, water-holding capacity, and thermal stability of thermogenic composite gels were enhanced by the direct introduction of gellan gum into soy protein isolate gels [8]. *Auricularia polytricha* polysaccharides formed a dense gel network by inducing enhanced intermolecular interactions between whey isolate proteins, which reduced the water mobility within the composite gels [9]. It evidenced the electrostatic interaction formed between OVA and kappa-carrageenan, which endowed the highest apparent viscosity and remained complex modulus [10].

Carboxymethyl cellulose sodium (CMC-Na), a common food additive, is a water-soluble cellulose derivative [11,12]. It draws considerable attention to CMC-Na because of its multifunctional role in the food industry, such as high thickening, strong water absorption, gelation, as well as good bulking abilities [13,14]. It reported that incorporating CMC-Na with soy protein isolate through glycosylation could improve the rheological properties and riboflavin delivery ability of soy protein isolate composite gels [15]. CMC-Na can affect the properties of protein gels. It reported that CMC-Na could reduce the pore size and increase the gel strength of fish scale collagen gels. CMC could also improve the functional properties of proteins, resist hydrolysis in the upper gastrointestinal tract, as well as encapsulate biologically active ingredients [16,17]. However, there are rare studies on the modulation of OVA gel properties by CMC-Na, especially combining glucono-δ-lactone induction with heat treatment.

Hence, this study investigated the effects of CMC-Na on the structural properties, water-holding capacity, textural properties, and water distribution of OVA gels induced by GDL and heat treatment. It aimed to discover the regulation for the rheological and textural properties of the OVA gels. The results may provide a new idea for the development of soft OVA gel foods with desirable textures and crowd.

## 2. Results and Discussion

### 2.1. Rheological Characteristics of OVA/CMC-Na Composite Gels

**Figure 1:**
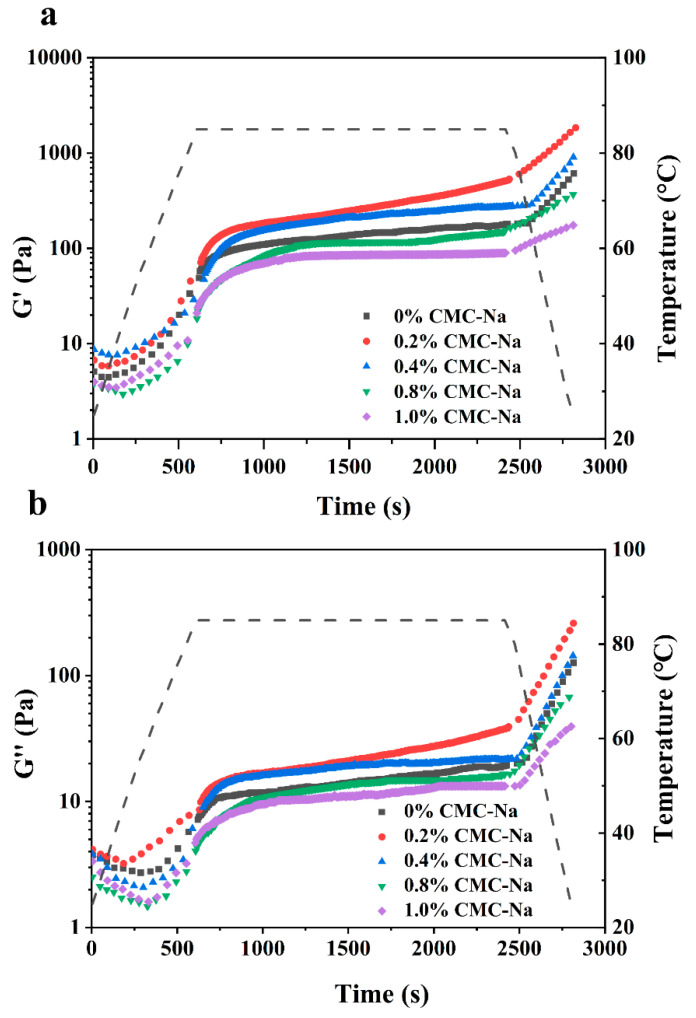
Storage moduli (**a**) and loss moduli (**b**) of OVA/CMC-Na composite gels with different CMC-Na contents during the temperature sweep.

To evaluate the effect of CMC-Na on the viscoelasticity of the OVA gels, the composite gels were subjected to temperature sweeps (25–85 °C) and the variation in the G′ and G″ (Figure 1). G′ and G″ represent the resilience and stickiness features, respectively. Tan δ, the ratio of G″ to G′, is applied to assess the viscoelastic strength of the gel system. It was found that GDL and heating treatment induced OVA and OVA/CMC-Na gelation. At the initial stage of heating (25–55 °C), the G′ and G″ of all composite gels decreased, which was related to the weakening of hydrogen bonding interactions in the composite gels. The phenomenon may also result from the steric hindrance and phase separation during GDL and heat treatment. During the heat temperature rising up to 85 °C, GDL released hydrogen protons and converted to gluconic acid, which caused the pH of the system to decrease and promoted the aggregation of OVA molecules. During the constant temperature phase, more sulfhydryl and disulfide bonds within OVA were exposed and rearranged. The intermolecular interactions were enhanced, which promoted the formation of a dense and elastic gel network. It was evidenced by the continued increase in G′ and G″ [18]. However, the subsequent cooling treatment (85–25 °C) promoted the hydrogen-bonding interactions between OVA and CMC-Na, which further enhanced the stability of the gel network. Consequently, the G′ and G″ of the gel samples continued to increase. Moreover, the final G′ increased and then decreased with the increase in CMC-Na addition, and Tan δ had an opposite trend. The OVA gel with 0.2% (*w*/*v*) CMC-Na addition had the largest G′ and the smallest tan δ (Table 1). These results indicate that moderate amounts of CMC-Na and OVA can form structurally solid composite gels under acid–heat co-treatment. However, when the CMC-Na addition was over 0.2% (*w*/*v*), the viscoelasticity of the composite gels was lower than that of the OVA gel, which was possibly due to the fact that the excess CMC-Na promotes the occurrence of phase separation under GDL and heat treatment conditions. A similar trend was observed in an OVA gel supplemented with carrageenan [19]. In summary, the modulation of the viscoelasticity of OVA/CMC-Na composite gels can be achieved by varying the amount of CMC-Na added.

### 2.2. FTIR of OVA/CMC-Na Composite Gels

**Figure 2:**
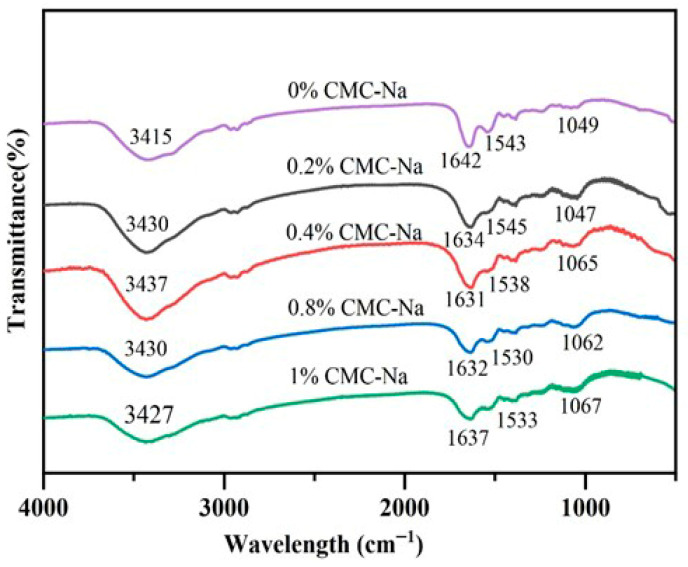
FTIR spectra of OVA/CMC-Na composite gels with different CMC-Na additions.

Figure 2 shows the FTIR results of CMC-Na/OVA composite gels with different CMC-Na concentrations. The pure OVA gel sample exhibited multiple characteristic absorption peaks: stretching vibration of -OH (3100 cm^−1^–3600 cm^−1^), stretching vibration of C=O (1600 cm^−1^–1700 cm^−1^), C-N stretching vibration, and N-H bending vibration (1500 cm^−1^–1600 cm^−1^) [20]. Although the FTIR spectra of the CMC-Na/OVA composite gel were similar to those of the pure OVA gel, some differences were observed. After the introduction of CMC-Na, the -OH stretching vibration peaks of the composite gels were shifted toward higher wave numbers, suggesting that the hydrogen bonding interaction between CMC-Na and OVA was enhanced. Moreover, the shift in the composite gel samples was most obvious at the addition of 0.4% CMC-Na. It suggested the strongest internal hydrogen bonding interactions. The enhanced hydrogen bonding interactions of composite gels were also noted after the introduction of dextran into acid-induced fava bean protein gels [21]. In the amide I band range (1600 cm^−1^–1700 cm^−1^), the characteristic peak of the pure OVA gel was 1642 cm^−1^, while the addition of CMC-Na shifted the characteristic peak toward the short wavelengths. It may be caused by the altered electrostatic interactions between CMC-Na and OVA. This phenomenon was consistent with previous findings [22,23].

### 2.3. Water-Holding Capacity of OVA/CMC-Na Composite Gels

**Figure 3:**
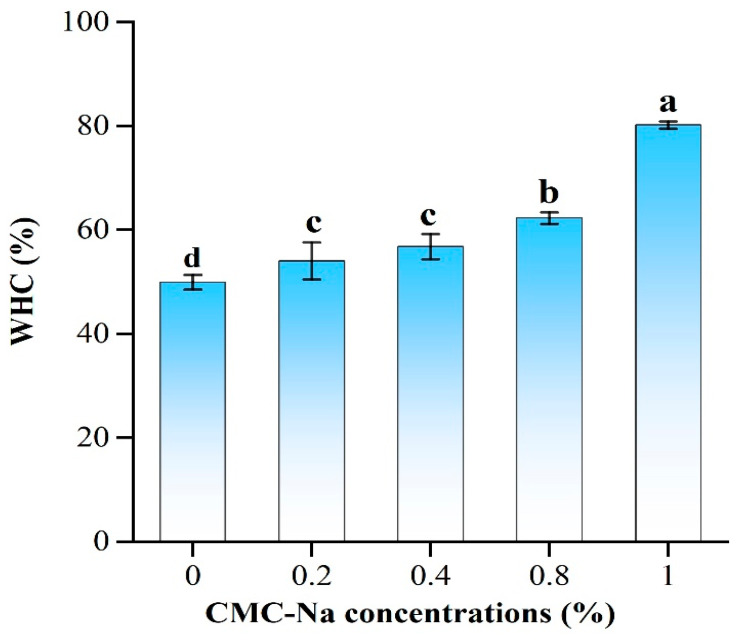
WHC of CMC-Na/OVA composite gels prepared with various CMC-Na additions. Different letters (a–d) represent significant differences in the same column (*p* < 0.05).

WHC is commonly employed to evaluate the ability of the gel network to trap water. As displayed in Figure 3, the presence of CMC-Na notably improved the WHC of the OVA/CMC-Na composite gels. When the concentration of CMC-Na was increased to 1%, the WHC of the composite gels increased from 49.93% to 80.19%. OVA/CMC-Na composite gels with high WHC show outstanding processing properties [24]. Similar reports showed that the addition of arabinoxylan significantly increased the WHC of GDL-induced casein gels [25,26]. The phenomenon can be attributed to two factors. Firstly, the proteins cross-link with the polysaccharides, enhancing the network structure of the composite gels. Additionally, the presence of numerous hydrophilic groups in CMC-Na binds to water and traps water molecules. However, the type of polysaccharide also affects the WHC of composite gels. It was reported that the addition of soybean soluble polysaccharides (SSPSs) reduced the WHC of GDL-induced soybean isolate protein (SPI) gels. This could be explained by the fact that the presence of SSPS weakened the hydrogen bonding interactions in the gel structure, leading to a loosening of the network structure of the composite gels and ultimately manifesting as a reduction in WHC [27,28].

### 2.4. Water Mobility of OVA/CMC-Na Composite Gels

**Figure 4:**
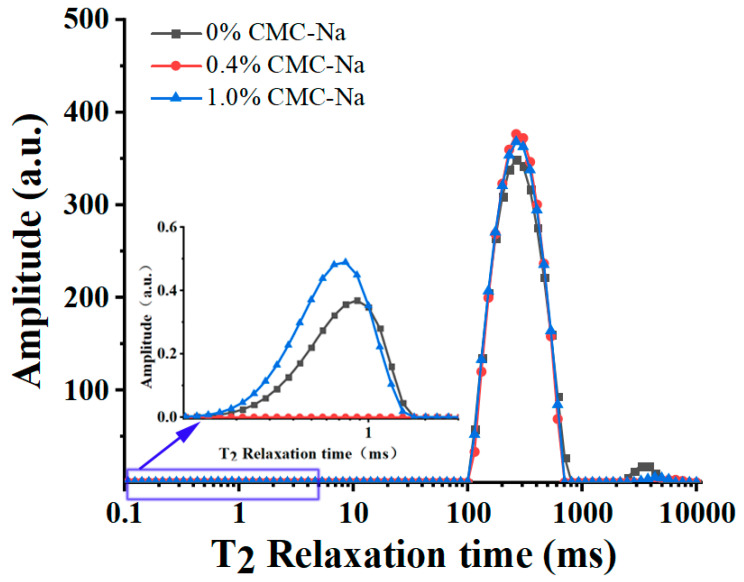
The effect of CMC-Na addition on T_2_ relaxation time distribution curves.

As shown in Figure 4, the T_2_ transverse relaxation time was determined by LF-NMR to analyze the effect of CMC-Na addition on the water distribution of the composite gels. Table 2 shows the percentage of relaxation peak areas of the composite gel samples with different additions of CMC-Na. The OVA gel had three relaxation peaks, representing bound water (T_21_, 0.1–10 ms), immobile water (T_22_, 100–1000 ms), and free water (T_23_, >1000 ms) [29]. Interestingly, the T_21_ relaxation peak of the composite gels disappeared after adding 0.4% CMC-Na. But the area percentage of the T_22_ relaxation peak increased, and the area percentage of the T_23_ relaxation peak decreased. It indicated that the content of the immobile water in the composite gel system increased, and the content of the free water decreased. Nevertheless, for OVA/CMC-Na composite gels with 1% CMC-Na, the T_21_ relaxation peak reappeared, which was accompanied by a continued increase and decrease in the area percentage of the T_22_ and T_23_ relaxation peaks, respectively. These results indicate that the introduction of CMC-Na was able to reduce water mobility, which is in line with the results of the water-holding capacity (Figure 3). Additionally, potato starch can inhibit water migration and improve the water-holding capacity of the composite gel system by enhancing the degree of binding of surimi to water molecules [30,31].

### 2.5. Texture Properties of OVA/CMC-Na Composite Gels

**Figure 5:**
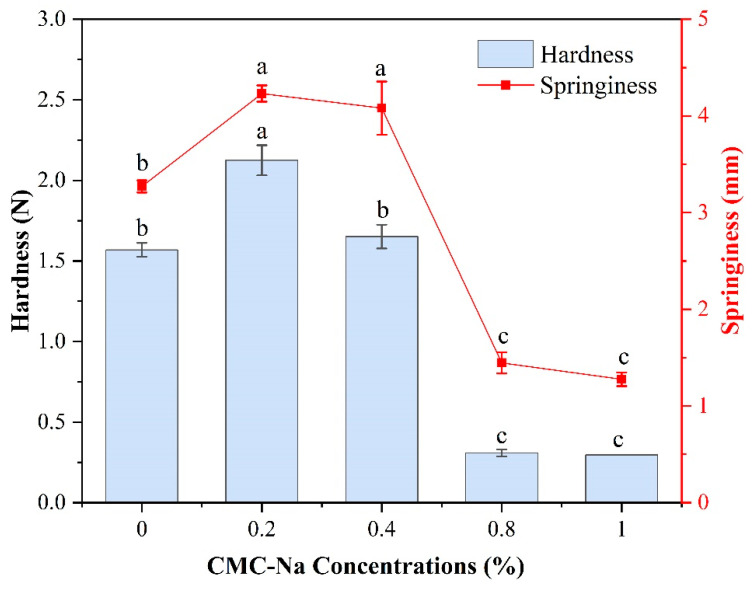
The hardness and springiness of CMC-Na/OVA composite gels prepared with various CMC-Na additions. Different letters represent significant differences (*p* < 0.05).

Texture measurements are commonly employed to evaluate the palatability, chewiness, and swallowability of gel foods. The textural features of OVA/CMC-Na composite gels can typically be indicated by their hardness and springiness. In this investigation, the hardness and springiness of the composite gels were considerably influenced by the different CMC-Na contents (Figure 5). With the increasing concentration of CMC-Na, the hardness and springiness of the composite gels increased and subsequently dropped. Furthermore, maximum hardness and springiness were recorded in the composite gels with 0.2% CMC-Na. In comparison to the 0.2% CMC-Na addition, the 0.8–1% CMC-Na addition decreased the hardness and springiness of the composite gels by more than 85%. The possible reason for this was that the interaction between the low concentration of CMC-Na and OVA was enhanced, and the composite gel network was filled, leading to improved textural properties [32]. Similar textural change was observed after the formation of complex gels with OVA by sesbania gum or xanthan gum [33]. As the concentration of CMC-Na further increases, the polymer formed by the cross-linking of CMC-Na with OVA hinders molecular aggregation and may induce the occurrence of phase separation, which weakens the composite gel network structure and ultimately leads to a significant reduction in hardness and springiness. In general, adding an appropriate amount of CMC-Na can significantly regulate the textural qualities of the OVA/CMC-Na composite gels.

### 2.6. Microstructure of OVA/CMC-Na Composite Gels

**Figure 6:**
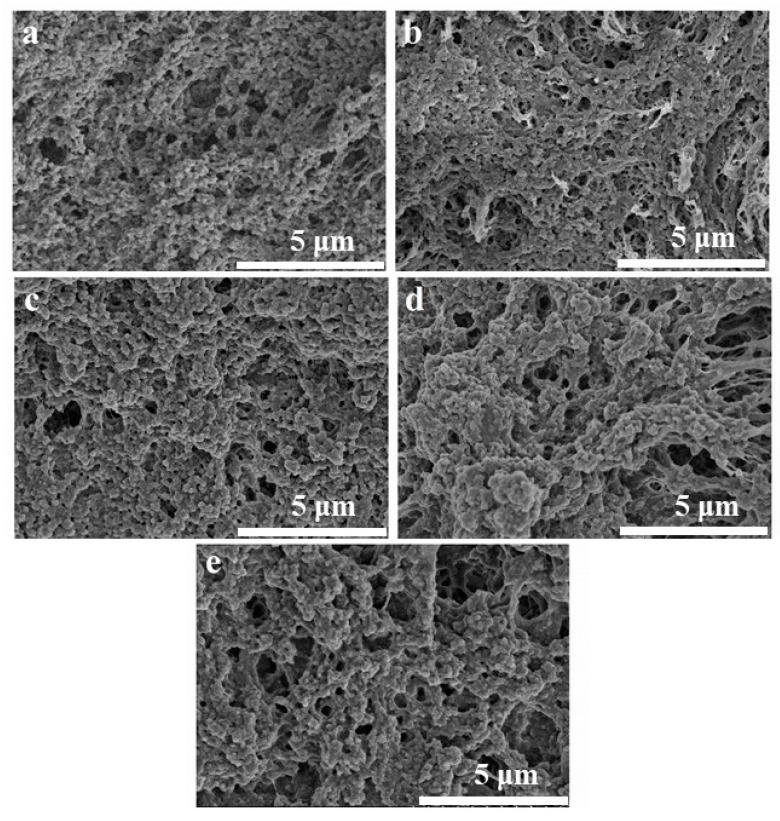
SEM images of OVA/CMC-Na composite gels with different CMC-Na addition ((**a**) 0%, (**b**) 0.2%, (**c**) 0.4%, (**d**) 0.8%, (**e**) 1%).

**Figure 7:**
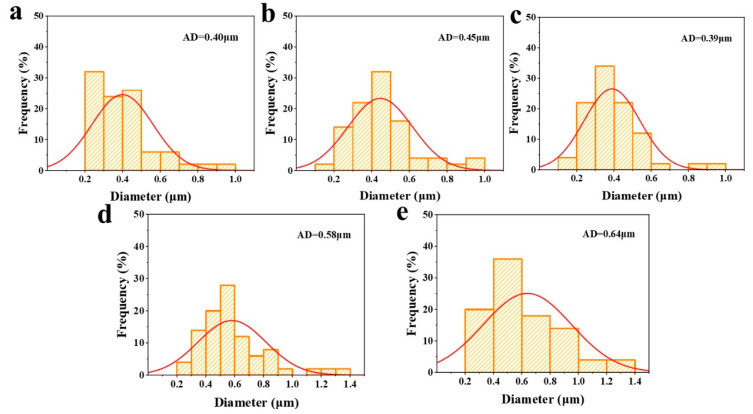
Pore size analysis from SEM images of composite gels with different CMC-Na additions ((**a**) 0%, (**b**) 0.2%, (**c**) 0.4%, (**d**) 0.8%, (**e**) 1%).

In Figure 6, the SEM images of the composite gels with different CMC-Na additions are demonstrated. The structure of the OVA gel induced by GDL and heat treatment was relatively loose with network pores of different sizes and disorganized distribution (Figure 6). However, the introduction of no more than 0.4% CMC-Na can improve the network structural densities of the composite gels, resulting in a smaller and more homogeneous distribution of network pores in the gels. It was probably due to the GDL-promoted interaction between OVA and CMC-Na, which formed a dense cross-linked network structure and reduced the network pore size. The pore size analysis agreed well with the SEM results (Figure 7). Furthermore, as the concentration of CMC-Na continually increased, excessive cross-linking within the OVA/CMC-Na composite gels resulted in the formation of a larger network aperture. It reduced the weakening of the structural strength of the system. This phenomenon was more obvious in the composite gel sample with 1.0% CMC-Na. The previous report showed that the introduction of 0.75% microcrystalline cellulose (MCC) into GDL-induced soybean isolate protein (SPI) gels resulted in the formation of larger protein aggregates and network pores [34]. The pore size and microstructure of OVA/CMC-Na composite gels agreed well with the texture properties.

## 3. Conclusions

The CMC-Na/OVA composite gels were prepared by GDL and heat treatment. The effects of CMC-Na on the structural, rheological, and textural properties of the composite gels were systematically studied. FT-IR spectroscopy confirmed that the hydrogen bonding interaction between CMC-Na and OVA improved the stability of the composite gels. The LF-NMR results showed that CMC-Na addition transformed the free water in the gel into immobile water, and effectively improved the binding ability of the gel with water molecules. Compared to 0.2% (*w*/*v*) CMC-Na addition, the 0.8–1% (*w*/*v*) CMC-Na decreased the hardness and springiness of the composite gels by more than 85%. The microstructure showed that the surfaces of the OVA gels were rough and porous. A low amount (<0.2%, *w*/*v*) of CMC-Na resulted in a smaller and more homogeneous distribution of the network with a low pore size distribution, while it displayed a larger network aperture and higher pore size distribution when a greater amount (>0.2%, *w*/*v*) of CMC-Na was added. The results suggested that CMC-Na could regulate the functional properties and microstructure of the OVA gels under GDL and heat treatment. The results provide theoretical support for the development and application of protein-based gel foods with desired development requirements.

## 4. Materials and Methods

### 4.1. Materials

Carboxymethyl cellulose sodium (CMC-Na) was purchased from Sinopharm Chemical Reagent Co., Ltd. (Shanghai, China). Ovalbumin (OVA) was purchased from Merck KGaA (Darmstadt, Germany) with a purity of 80%. Glucono-δ-lactone (GDL) was obtained from Yuanye biological technology Co., Ltd. (Shanghai, China). All other reagents were of analytical reagent grade. The solutions were prepared using ultrapure water, which was obtained from Sichuan Youpu Ultrapure Technology Co., Ltd. (Sichuan, China).

### 4.2. Preparation of Composite Gels

The preparation of CMC-Na/OVA composite gels induced by GDL and heat treatment was referenced in a previous study [35]. Briefly, 6% (*w*/*v*) OVA solution was first prepared by dissolving 6 g OVA in 100 mL ultrapure water by stirring for 2 h at 20 °C. The OVA solution was then added with various amounts (0.2%, 0.4%, 0.8%, 1.0%, *w*/*v*) CMC-Na and 5% (*w*/*v*) GDL. Finally, the mixtures were heated in a water bath at 85 °C for 0.5 h. After cooling to room temperature, the mixture was gelled at 4 °C for 24 h to generate CMC-Na/OVA composite gels. Furthermore, OVA gel without CMC-Na was prepared as control samples as described above.

### 4.3. Rheological Characteristics

A rheometer (DHR-2, TA Instruments, New Castle, DE, USA) equipped with a parallel plate (d = 40 mm) was employed to conduct the rheological characterization of CMC-Na/OVA composite gels. For temperature sweeps of the composite gel samples, CMC-Na/OVA mixture with GDL was prepared by the method above. The mixture (1.5 mL) was placed on the sample stage. The distance between the parallel plate and the sample stage was 1 mm. The excess mixture on the stage was then scraped off, and a layer of silicone oil was applied between the sample stage and the parallel plate to reduce water evaporation. Temperature sweep tests were carried out with a strain of 1% and a frequency of 1 Hz. The storage modulus (G′), loss modulus (G″), and loss factor (Tanδ) were recorded. During the experiment, the temperature was first increased from 25 °C to 85 °C with a temperature change rate of 5 °C/min and then maintained at 85 °C for 30 min. Finally, the samples were then cooled down to 25 °C at the same speed.

### 4.4. Fourier Transform Infrared Spectroscopy (FTIR)

The spectral characteristics of the CMC-Na/OVA composite gels were recorded using a Fourier transform spectrophotometer (Perkin–Elmer Corporation, Norwalk, CT, USA) as previously described [36]. Specifically, freeze-dried gels were mixed and triturated with dried KBr (1:50) and pressed into thin slices. FTIR spectra were obtained by accumulating 32 scans in the range of 4000–500 cm^−1^ with a resolution 4 cm^−1^.

### 4.5. Water-Holding Capacity (WHC) Measurement

The WHC of the CMC-Na/OVA composite gels was measured by centrifugation. Approximately 3 g of the gel samples were weighed into a centrifuge tube and centrifuged (6500 rpm, 15 min) at 4 °C. The supernatant was then poured out. The water was removed from the surface of the gel sample using filter paper. Finally, the total mass of the precipitate and centrifuge tube was weighed. The WHC of the composite gels was calculated according to the following formula:

(1) $$\mathrm{WHC}\;\left(\%\right)=\frac{W_{2}-W_{0}}{W_{1}}\times 100$$

where W_0_ is the mass of the centrifuge tube (g), W_1_ is the mass of the gel samples before centrifugation (g), and W_2_ is the total mass of the gel samples and the centrifuge tube with the supernatant removed (g).

### 4.6. Low Field Nuclear Magnetic Resonance (LF-NMR)

The moisture distributions of the CMC-Na/OVA composite gels were measured using an LF-NMR instrument (NMI20-015V-1, Niumag Co., Ltd., Shanghai, China) [37,38]. In brief, about 3 g of gel samples was taken in a glass vial (d = 5 mm), which was then placed in an NMR glass tube, and the spin–spin relaxation time (T_2_) was measured with a Carr–Purcell–Meiboom–Gill (CPMG) sequence. The LF-NMR instrument was operated at 32 °C with the following measurement conditions: waiting time (TW) 2000 ms, radio frequency delay time (RFD) 0.080 ms, echo time (TE) 1.000 ms, and number of echoes (NECH) 10,000.

### 4.7. Texture Tests

Before the texture tests, CMC-Na/OVA composite gel (height = 22 mm) was prepared as described above. The textural properties of the composite gels were evaluated using the TPA mode of a texture analyzer (TMS-Pro 300, FTC, Sterling, VA, USA) with a P/0.5 cylindrical probe [39]. The test parameters were as follows: test rate 2 mm/s, morphology 50%, and trigger force 0.1 N. Each group of composite gel samples was measured five times.

### 4.8. Scanning Electron Microscopy (SEM)

The microscopic morphology of the CMC-Na/OVA composite gels was observed by a SEM (Regulus 8220, Hitachi, Japan) with the previous method [40]. Prior to the experiments, the CMC-Na/OVA gels were cut into thin slices and freeze-dried for 72 h. Afterwards, the freeze-dried CMC-Na/OVA gels were fixed with conductive adhesive and sprayed with gold powder. The microscopic morphology was observed under an accelerating voltage of 3.0 kV and a magnification of 10.0 k.

### 4.9. Statistical Analysis

The data were reported as mean ± standard deviation. SPSS software (version 25.0, Chicago, IL, USA) and Origin 2021Pro were utilized for data analysis and graphical plotting, respectively. Furthermore, Duncan’s test was employed for significance analysis, and *p* < 0.05 represents a significant difference.

## Figures and Tables

**Table 1:** Effect of CMC-Na addition on G′, G″, and Tanδ of OVA/CMC-Na composite gels.

CMC-Na Addition (%)	Final G′ (Pa)	Final G″ (Pa)	Final Tanδ
0	609.34	126.07	0.21
0.2	1840.52	260.52	0.14
0.4	904.01	143.67	0.16
0.8	366.22	67.17	0.18
1.0	180.00	39.41	0.22

**Table 2:** Effect of CMC-Na addition on the water distribution of OVA/CMC-Na composite gels.

CMC-Na (%)	P_T21_ (%)	P_T22_ (%)	P_T23_ (%)
0	0.05 ± 0.05 ^a^	98.12 ± 0.66 ^b^	1.85 ± 0.65 ^b^
0.4	0.00 ± 0.00 ^a^	99.52 ± 0.47 ^a^	0.81 ± 0.37 ^ab^
1.0	0.14 ± 0.02 ^a^	99.67 ± 0.29 ^a^	0.35 ± 0.26 ^a^

Note: Different lowercase letters mean significant differences in water distribution between gel samples with different CMC-Na additions (*p* < 0.05).

## Data Availability

The original contributions presented in this study are included in the article. Further inquiries can be directed to the corresponding author.
